# A comparison of sample preparation methods for extracting volatile organic compounds (VOCs) from equine faeces using HS-SPME

**DOI:** 10.1007/s11306-017-1315-7

**Published:** 2018-01-04

**Authors:** Rachael Hough, Debra Archer, Christopher Probert

**Affiliations:** 1 Department of Cellular and Molecular Physiology, University of Liverpool, Liverpool, UK; 2 Department of Epidemiology and Population Health, University of Liverpool, Liverpool, UK

**Keywords:** Volatile organic compounds, GC-MS, SPME, Faeces, Equine

## Abstract

**Introduction:**

Disturbance to the hindgut microbiota can be detrimental to equine health. Metabolomics provides a robust approach to studying the functional aspect of hindgut microorganisms. Sample preparation is an important step towards achieving optimal results in the later stages of analysis. The preparation of samples is unique depending on the technique employed and the sample matrix to be analysed. Gas chromatography mass spectrometry (GCMS) is one of the most widely used platforms for the study of metabolomics and until now an optimised method has not been developed for equine faeces.

**Objectives:**

To compare a sample preparation method for extracting volatile organic compounds (VOCs) from equine faeces.

**Methods:**

Volatile organic compounds were determined by headspace solid phase microextraction gas chromatography mass spectrometry (HS-SPME-GCMS). Factors investigated were the mass of equine faeces, type of SPME fibre coating, vial volume and storage conditions.

**Results:**

The resultant method was unique to those developed for other species. Aliquots of 1000 or 2000 mg in 10 ml or 20 ml SPME headspace were optimal. From those tested, the extraction of VOCs should ideally be performed using a divinylbenzene-carboxen-polydimethysiloxane (DVB-CAR-PDMS) SPME fibre. Storage of faeces for up to 12 months at − 80 °C shared a greater percentage of VOCs with a fresh sample than the equivalent stored at − 20 °C.

**Conclusions:**

An optimised method for extracting VOCs from equine faeces using HS-SPME-GCMS has been developed and will act as a standard to enable comparisons between studies. This work has also highlighted storage conditions as an important factor to consider in experimental design for faecal metabolomics studies.

**Electronic supplementary material:**

The online version of this article (10.1007/s11306-017-1315-7) contains supplementary material, which is available to authorized users.

## Introduction

The equine hindgut contains a complex and diverse community of microorganisms (microbiota). The microbiota is essential for the breakdown of fibre into volatile fatty acids (VFA), providing a major source of energy (Bergman 1990). Disturbance to the hindgut microbiota can be detrimental to equine gastrointestinal health resulting in colic (Daly et al. 2012) and laminitis (Milinovich et al. 2007). Metabolomics provides a tool for characterising the functionality of the gut microbiota and therefore is a useful means for the study of gut dysbiosis (Marcobal et al. 2013).

Volatile organic compounds (VOCs) are a large group of carbon-containing molecules, which may be of biological or synthetic origin. The low molecular weight and high vapour pressure of VOCs allows them to enter the gaseous phase at room temperature; contributing to the odour of faeces, urine, breath, saliva, blood and sweat. These compounds may be generated by physiological processes from the host or by its microbiota (Amann et al. 2014). VOC analysis of faeces provides a simple approach to understanding functional changes of the microbiota of the distal intestine. The faecal VOC profiles of horses have been found to alter in response to dietary supplementation, from the ingestion of probiotics and when suffering from gastrointestinal disease (colic) (Turner et al. 2013; Ishizaka et al. 2014; Proudman et al. 2014).

Headspace, solid phase microextraction gas chromatography mass spectrometry is a rapid and economical technique that has been used to characterise the faecal metabolome in human subjects and many species including ruminants, poultry and the horse (Garner et al. 2007, 2008; Stavert et al. 2014). The use of SPME to extract analytes from biological samples is advantageous as it does not require the addition of organic solvents (Arthur and Pawliszyn 1990). GCMS is one of the most widely used platforms for the study of metabolomics yet an optimised method has yet to be studied for equine faeces. The preparation steps of samples for metabolomics are vital for achieving optimal results and consistent data in the later stages and a standardised method will allow the comparison of results between laboratories. It has previously been demonstrated that vial volume, SPME fibre coating and mass of faecal material have an effect on the number and abundance of VOCs in human and murine faecal samples (Reade et al. 2014). This is not surprising as faecal VOC profiles differ between species (Saric et al. 2008). In addition, the optimal preparation steps differed between human and murine faeces (Reade et al. 2014). Therefore, it is expected that a modified method for GCMS VOC profiling of equine faeces is needed.

The sample preparation factors investigated in this work were: faecal sample mass, SPME fibre type, headspace vial volume and the reproducibility of the optimised method proposed. Sample storage for varying time and temperatures were also investigated in one individual pony.

## Methods

### Animals and sample collection

#### Part (A)

Faecal samples were collected in June 2015 from four horses immediately after spontaneous defaecation. The horses were Thoroughbred and Irish sport horse crossbreeds, demographic information for each horse is listed in Supplementary Table 1. Samples were transported on ice and transported to the laboratory where they were frozen at − 20 °C within 0.5–3 h and were stored until analysis. All horses were housed on the same premises (Phillip Leverhulme Equine Hospital, Leahurst Campus, University of Liverpool) and were maintained under the same conditions: living out at pasture all year round with a diet supplemented with hay during the winter. The horses were stabled for periods during the daytime to be handled by students. During this time horses had access to ad libitum hay. Each horse was treated for intestinal parasites with ivermectin and praziquantel 4 weeks previously.

#### Part (B)

A faecal sample was collected from a mixed-breed pony mare (P1) on private premises in May 2016 to investigate the effect of storage on faecal VOCs. The sample was collected after spontaneous defaecation and immediately transported to the laboratory (< 2 h). Samples were stored at either − 20 °C (n = 9) or − 80 °C (n = 9) until analysis. The pony (aged 16 years) was maintained at pasture all year round and received a diet supplemented with hay during the winter. At the time of collection, a faecal egg count of 0.0. eggs per gram was recorded and praziquantel had been administered 4 weeks previously. A summary of the collection and division of samples from Part A and Part B is shown in Table 1.

**Table 1:** A faecal sample was collected from Horse 1, 2, 3, 4 and Pony 1 to compare methods for extracting VOCs using HS-SPME-GCMS

Experimental factor	Horse 1	Horse 2	Horse 3	Horse 4
	Number of technical replicates			
Sample mass				
100 mg	3	3	3	3
1000 mg	3*	3*	3*	3*
2000 mg	3	3	3	3
SPME fibre type				
DVB-CAR-PDMS	3*	3*	3*	3*
CAR-PDMS	3	3	3	3
Headspace vial volume				
10 ml	3*	3*	3*	3*
20 ml	3	3	3	3

	Pony 1			
Storage				
Fresh	3			
− 20 °C 1 week	3			
− 20 °C 6 months	3			
− 80 °C 1 week	3			
− 80 °C 6 months	3			

Experimental conditions and the numbers of technical replicates are listed. Technical replicates marked with * indicate these were included in an additional analysis to test the reproducibility of the method

### Head space-solid phase micro extraction (HS-SPME)

VOCs were extracted from the headspace of faeces when the SPME fibre was exposed to the headspace of a 10 ml or 20 ml glass vial (Supelco, Dorset, UK) as programmed using Combi Pal auto-sampler (CTC Analytics, Switzerland). Vials were incubated at 60 °C for 30 min, followed by a 20 min extraction at 60 °C. The SPME fibres used were divinylbenzene-carboxen-polydimethysiloxane (DVB-CAR-PDMS) 50/30 µm (1 cm) or carboxen-polydimethysiloxane (CAR-PDMS) (85 µm) (Sigma-Aldridge, Dorset, UK). The SPME fibres were pre-conditioned in accordance with manufacturer’s instructions before use.

### GC-MS conditions

GC-MS analysis was carried out using a Perkin Elmer Clarus 500 GC/MS Quadruple bench top system (Beaconsfield, UK). Separation of VOCs was performed in the Zebron ZB-624 GC column with an inner diameter of 0.25 mm, length of 60 m and a film thickness of 1.4 µm (Phenomenex, Macclesfield, UK). Helium of 99.996% purity was used as a carrier gas, set at a flow rate of 1 ml/min (BOC, Sheffield, UK). SPME fibre desorption temperature and time were 220 °C and 5 min, respectively. The GC oven was initially set at 40 °C and held for 1 min before a ramp to 220 °C at a rate of 5 °C per min and held at 4 min (total run time of 41 min). The MS was operated in electron impact ionization EL + mode, scanning ion mass fragments from 10 to 300 *m*/*z* with an inter-scan delay of 0.1 s and a resolution of 1000 FWHM (Full Width at Half Maximum). Laboratory air was sampled frequently to rule out possible contaminants from the analysis. Blank vials were tested between samples to ensure VOCs were originating from faeces and to prevent a carry-over of VOCs on the SPME fibre between samples. Three commercially available standards of compounds putatively identified in this study (benzaldehyde, 2-pentanone and indole) were run and the data processed in the same way as experimental data to validate compound identification towards MSI level 1 (Sumner et al. 2007). Internal standards were not used for this non-targeted study because of the limited knowledge of compounds emitted from the equine faecal metabolome.

### Faecal mass optimisation

Triplicates of 100 mg (mean and SEM) (105.9 ± 1.3 mg), 1000 mg (1003.0 ± 4.1 mg), 2000 mg (2005.8 ± 5.1 mg) of faeces from each horse from Part A were divided into 10 ml vials to determine the number and abundance of VOCs. The SPME fibre DVB-CAR-PDMS was used to extract VOCs before injection into the GC oven.

### SPME fibre type

Two different fibre coatings were tested in order to determine whether the type of fibre coating used in SPME-GC-MS analysis has an effect on the VOC profile. The SPME fibre coatings chosen were CAR-PDMS and DVB-CAR-PDMS. Masses of 1000 mg (1008.9 ± 3.3 mg) were placed into six 10 ml vials for each horse from Part A. Three replicates were assigned to the CAR-PDMS fibre coating group and 3 replicates for the DVB-CAR-PDMS group. The samples then underwent HS-SPME-GC-MS analysis.

### Headspace volume

Vials of 10 and 20 ml were chosen in order to compare 1000 mg of faeces. Three 10 ml and three 20 ml vials containing 1000 mg (mean and SEM, 1003.9 ± 2.3 mg) of faeces from each horse from Part A underwent HS-SPME-GC-MS analysis using a DVB-CAR-PDMS fibre to extract VOCs.

### Technical replicates

A total of nine technical replicates of 1000 mg of faeces were analysed from horses (H1, H2, H3 and H4) from Part A. All samples were contained in 10 ml vials and VOCs were extracted using the SPME fibre DVB/CAR/PDMS. In order to assess the variation across technical replicates, statistical analysis was performed.

### Time and temperature of storage

Aliquots of faeces (1008 ± 0.6 mg, mean and SEM) collected from pony P1 (Part B) were placed into headspace vials. The samples were stored for 1 week, 6 and 12 months at − 20 or − 80 °C before analysis. Three technical replicates of each storage condition were analysed. A DVB-CAR-PDMS fibre was used to extract VOCs before injection into the GC oven. Three fresh aliquots of the faecal sample collected underwent HS-SPME-GCMS within 2 h of defecation to compare against frozen samples.

### Data processing

The data was processed using Automated Mass Spectral Deconvolution System (AMDIS-version 2.71, 2012) and the National Institute of Standards and Technology (NIST) mass spectral library (version 2.0, 2011) to putatively identify VOCs. The R package Metab (Aggio et al. 2011) was used to align the data. All samples were analysed in triplicate, an average was taken of the replicates at this stage and taken forward for data analysis to keep random errors to a minimum. Statistical analysis was performed in R version 3.1.2 and using the online software tool Metaboanalyst 3.0 (Xia et al. 2012).The data was filtered by removing VOCs that were not present in 50% of samples within at least one of the experimental conditions being compared. Missing values were imputed with half minimum values from each data matrix. The abundance data were normalised using log transformation (general logarithm) and group means compared using an independent t-test or one-way ANOVA without interactions where appropriate. Pair-wise comparisons were made by Tukey’s HSD test, followed by Bonferroni correction. Fisher’s exact test, followed by Bonferroni correction was performed to assess for statistical significance in absence or presence of compounds between conditions. All *p* values of less than 0.05 were considered to be significant. Principal component analysis (PCA) and dendrograms (hierarchical clustering) were constructed to visually compare VOC profiles.

## Results

### Faecal mass

**Fig. 1:**
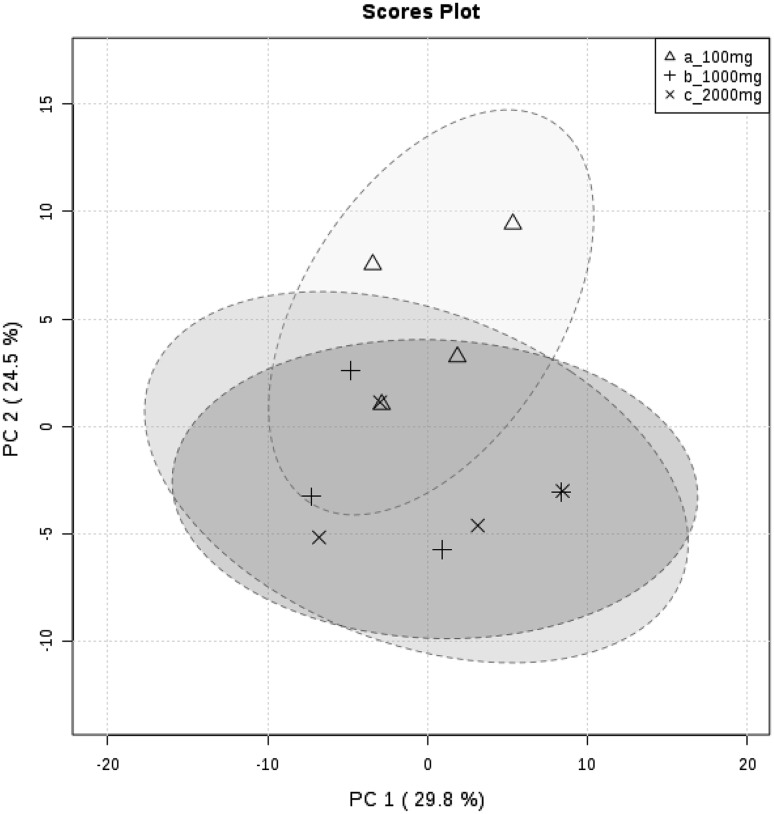
A PCA of the VOC profiles of 100, 1000 and 2000 mg of horse faeces analysed by HS-SPME-GCMS. Grouped samples are contained within a 95% confidence interval

The mean (± SD) number of VOCs detected from each sample mass were 59 (+ 9.5), 79 (+ 8.8) and 80 (+ 4.8) for 100, 1000 and 2000 mg, respectively. There were 13 VOCs (Supplementary Table 2) that were exclusive to 1000 and 2000 mg. All compounds detected were present in at least one sample of 1000 mg whereas three compounds (butanoic acid, 3-methyl-, propyl ester, propanoic acid, 2-methyl-, acetic acid) were missing from 2000 mg samples. One compound (2-decanone), was significantly lower in abundance in 100 mg than 1000 and 2000 mg (*p* < 0.01, ANOVA, Tukey’s HSD test and Bonferroni corrected). The coefficient of variation (CV) of VOC peak area was calculated for VOCs shared between replicated of each sample mass. Coefficient of variation values were 1.6–13, 1.1–14.4, 0.8–12.5% for 100, 1000 and 2000 mg, respectively. A full list if CV values for each VOC are shown in Supplementary Table 3. A PCA is shown in Fig. 1 which represents the VOC profiles of each sample mass. A list of scores for PC1 and PC2 is supplied in Supplementary Table 4.

### SPME fibre type

**Fig. 2:**
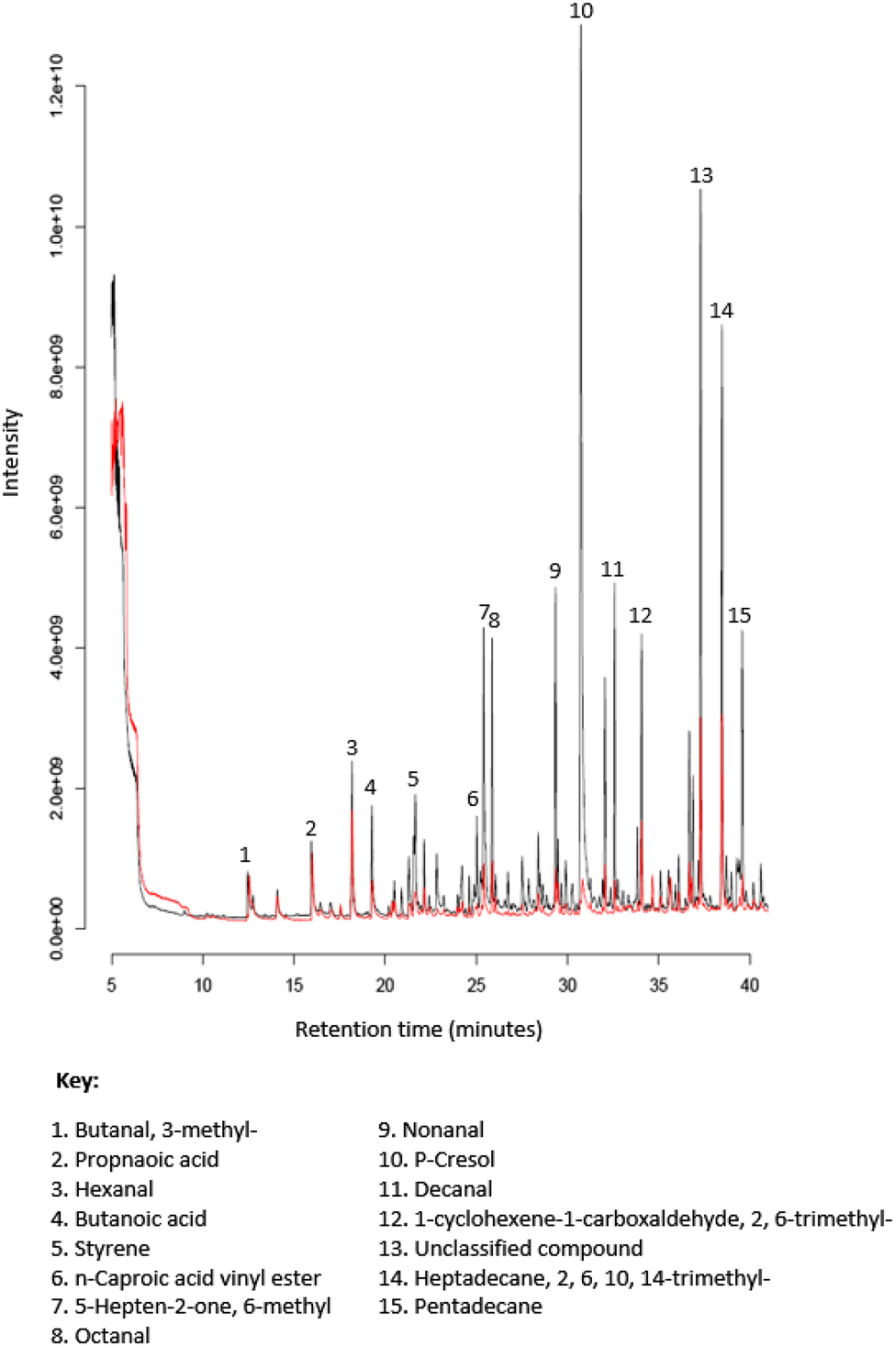
An overlay of chromatograms generated from the HS-SPME-GCMS analysis of faeces of horse 2 (H2). The black trace represents the DVB-CAR-PDMS fibre and the red is CAR-PDMS

Mean (± SD) number of compounds detected by the CAR-PDMS SPME fibre was 52 (± 11.2) and DVB-CAR-PDMS was 78 (± 7.8). Significantly more VOCs were detected with a DVB-CAR-PDMS fibre than from a CAR-PDMS fibre (*p* < 0.01, *t* test, Bonferroni corrected). 21 compounds were exclusive to the DVB-CAR-PDMS SPME fibre and one compound was exclusive to the CAR-PDMS SPME fibre. A list of compounds exclusive to each fibre is in Supplementary Table 5. One compound (benzaldehyde) was significantly greater in abundance in samples exposed to the DVB-CAR-PDMS SPME fibre than the CAR-PDMS SPME fibre (*p* < 0.05, ANOVA, Bonferroni corrected). A chromatogram overlay was generated for the DVB-CAR-PDMS and CAR-PDMS fibres for one of the horses (H2) and is shown in Fig. 2.

### Headspace vial volume

The mean (± SD) number of VOCs was 83 (± 9.6) and 78 (± 9.3) in 10 and 20 ml vials, respectively. Differences in mean VOC numbers between 10 and 20 ml vials were not significantly different from each other (*p* = 0.48, *t* test, Bonferroni corrected). Two compounds (propanoic acid, 2-methyl- and acetic acid, methyl ester) were exclusive to 20 ml vials and four compounds (propanoic acid, 2-methyl-, methyl ester, 2-hexanone, propanoic acid, 2-methyl-, propyl ester, 1-nonanol) were detected from 10 ml vials only. There was no significant difference in the abundance of any compounds shared between 10 and 20 ml vials (*p* > 0.05, *t* test, Bonferroni corrected).

### Technical replicates

**Fig. 3:**
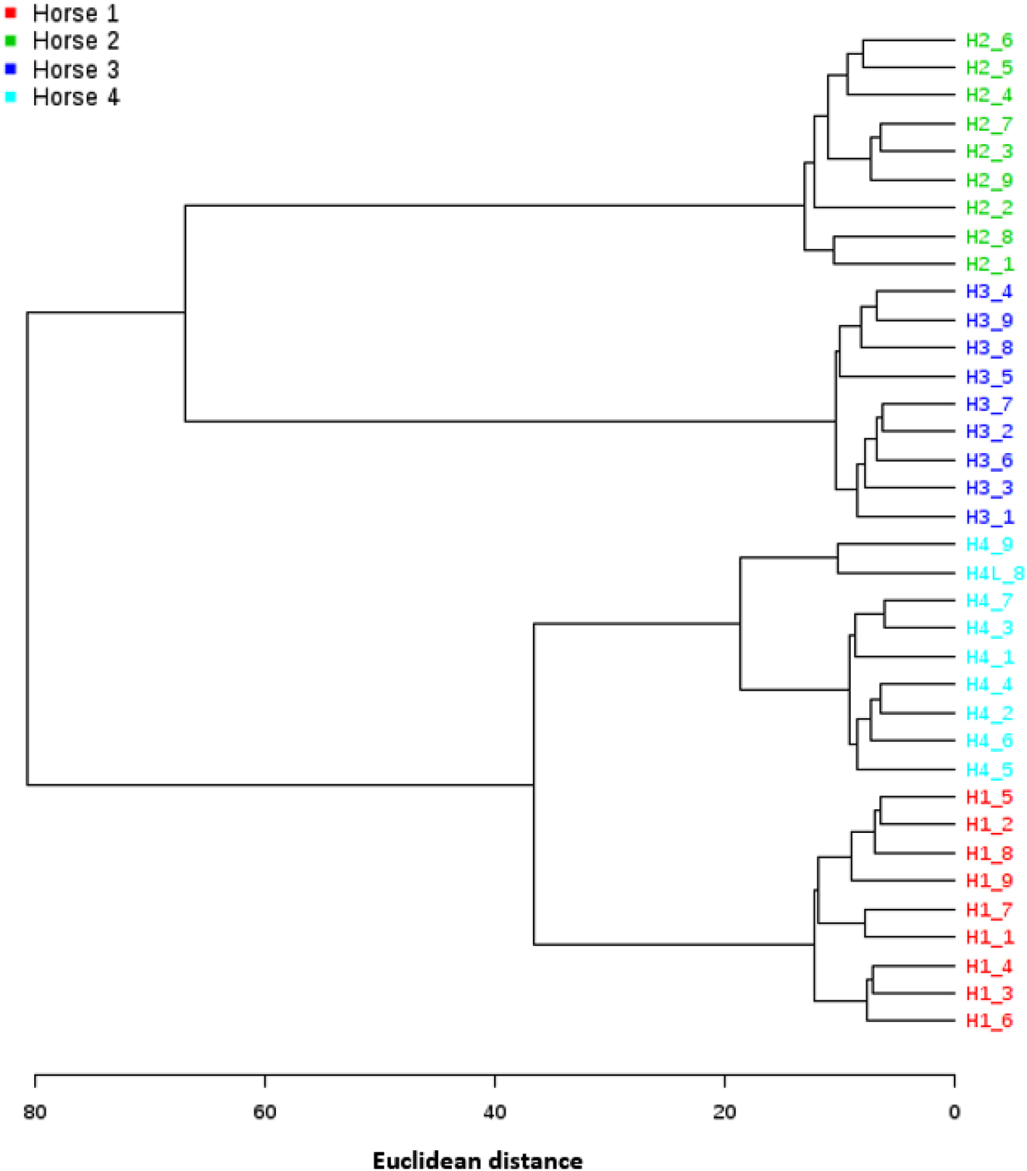
A dendrogram constructed using the elucidean distance and ward clustering algorithm. Technical replicates of 1000 mg of faeces from four horses, analysed by HS-SPME-GCMS. VOCs were extracted using a DVB-CAR-PDMS SPME fibre

The CV was performed on the peak areas of VOCs shared across nine technical replicates of 1000 mg of faeces for each horse. The CV for each VOC peak area and the mean numbers of VOCs detected in each horse are in Supplementary Table 6. To summarise the CV for VOC peak area from H1 ranged between 0.7 and 8.6, 1–11% for H2, 0.9–8.3% for H3 and 0.7 to 9.6% for H4. A cluster analysis was performed based on the VOC profiles of all technical replicates and is shown in Fig. 3.

### Time and temperature of storage

**Fig. 4:**
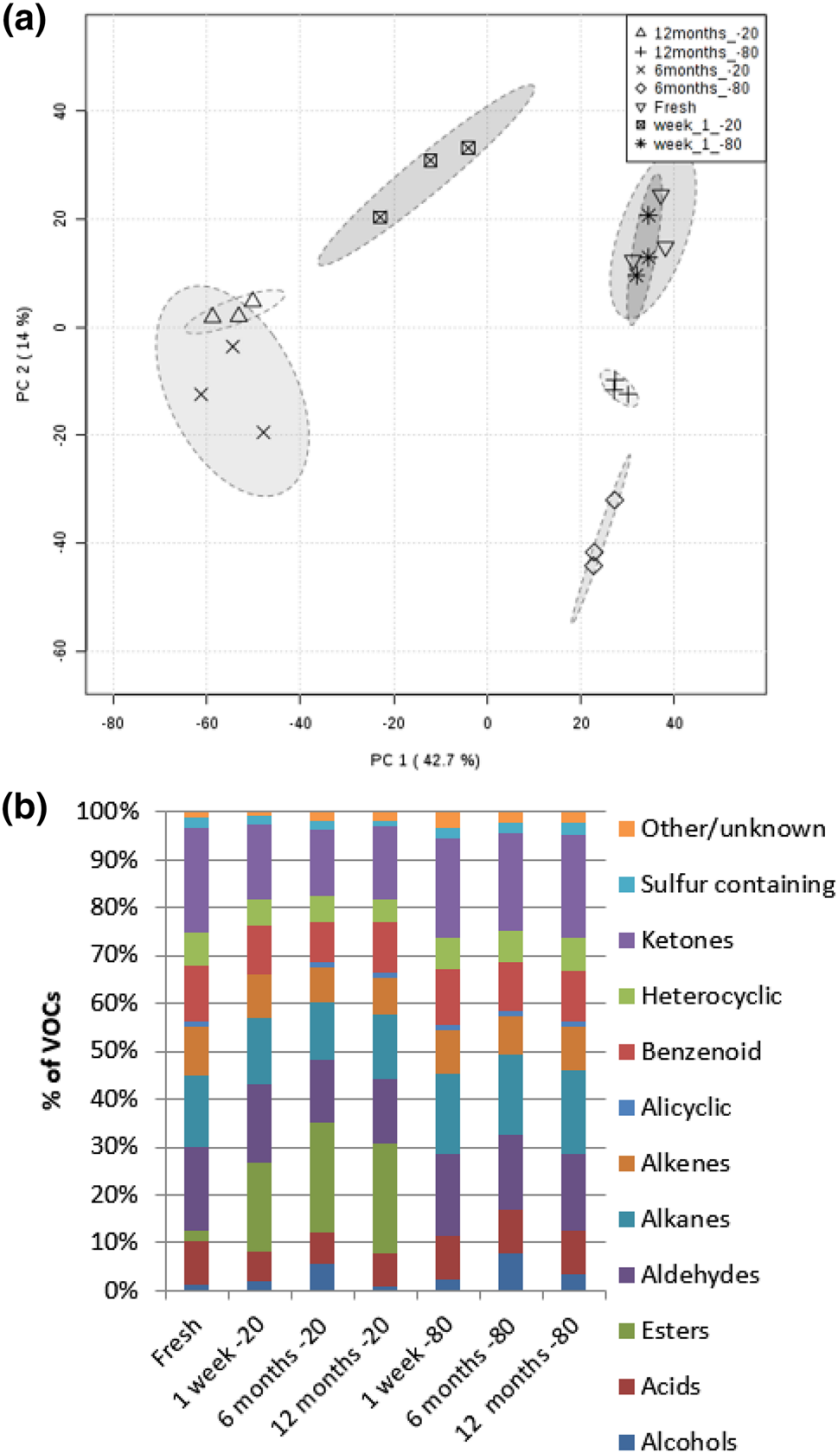
**a** A PCA of the VOC profiles of an equine faecal sample analysed by HS-SPME-GCMS 2 h post collection, after storage for 1 week, 6 and 12 months at − 20 and − 80 °C. 3 **b** A stack plot of the chemical classes of compounds found in each storage condition

The mean number of VOCs identified in the fresh faecal sample, storage after 1 week, 6 months and 12 months at − 20° and − 80° are shown in Table 2.The percentages of VOCs shared between fresh and stored samples are also shown in Table 2. A PCA of the VOC profiles and a stack plot of the chemical classes of compounds found in each condition are shown in Fig. 4. A list of scores for PC1 and PC2 is supplied in Supplementary Table 7.

**Table 2:** A table of the mean number of VOCs and percentages of VOCs shared between an equine faecal sample analysed by HS-SPME-GCMS 2 h post collection, after storage for 1 week at − 20 and − 80 °C and after 6 months at − 20 and – 80 °C

Storage conditions (°C)	Mean (± SD) numbers of VOCs
Fresh	79 (± 1.5)
1 week at − 20	99 (± 1.5)
1 week at − 80	82 (± 1.5)
6 months at − 20	95 (± 3.6)
6 months at − 80	80 (± 0.6)
12 months at − 20	93 (± 2.5)
12 months at − 80	80 (± 2.6)

Storage conditions (°C)	Percentage (%) of VOCs shared
Fresh and 1 week at − 20	79
Fresh and 6 months at − 20	65
Fresh and 12 months at − 20	64
Fresh and 1 week at − 80	91
Fresh and 6 months at − 80	84
Fresh and 12 months at − 80	84

## Discussion

### Sample mass

A mass of 100 mg of faeces produced fewer VOCs than 1000 or 2000 mg. Masses of 1000 and 2000 mg showed very little variation between them and therefore 1000 mg may be considered an optimal mass for HS-SPME-GCMS of equine faeces. Masses greater than 2000 mg were not investigated as little difference was seen between 1000 and 2000 mg. It is likely that saturation of the SPME fibre or over-loading of the instrument has started to occur (Ng et al. 2012). The PCA plot (Fig. 1) accounts for 54.3% of the variance in the data set. Toluene, butanoic acid, 3-methyl-, ethyl ester and butanoic acid, 2-methylbutyl ester were among the VOCs most responsible for the variation seen in PC1. For PC2 styrene, butanoic acid, ethyl ester, d-limonene were VOCs most responsible for variation. A number VOCs with high scoring PCs were also found to be exclusive to 1000 and 2000 mg samples (Supplementary Table 2).

### SPME fibre type

A greater number of VOCs was obtained when using DVB-CAR-PDMS fibre, rather than CAR-PDMS alone. The use of multiple fibre coatings increases the diversity of VOCs obtained (Dixon et al. 2011; Reade et al. 2014). The choice of fibre coating is an important factor affecting SPME as there is not a single fibre coating suited to all analytes. A DVB coating is mainly mesoporous, has a trapping range of C6-C15 and because of these properties is more suited to extracting medium and high molecular weight compounds (Mani 1999; Gianelli et al. 2002). Whereas a CAR coating is microporous, has a trapping range of C2–C12 and is more suited to low molecular weight compounds (Mani 1999; Gianelli et al. 2002). However, a more marked variation in VOC diversity between CAR-PDMS and DVB-CAR-PDMS was observed in the present work than in human faeces (Couch et al. 2013). Therefore, the use of DVB and CAR fibre coatings, combined with PDMS, appears to yield the best results for equine faeces: this is supported by the findings of Bianchi et al. when studying short chain fatty acids from an in vitro colonic fermentation model (Bianchi et al. 2011). Therefore, a combination of the two coatings results in a net increase in diversity of compounds and offers a wider range of extraction. Comparisons made between SPME and other extraction techniques including direct thermal desorption (Cavalli et al. 2003) and purge and trap (Povolo and Contarini 2003) have shown to yield different VOCs. Alternatives to SPME could yield a greater library of VOCs and should be considered for future method development.

### Headspace vial volume

The volume of the vial headspace containing 1000 mg of faeces did not have an effect on the number of VOCs obtained from the headspace of faeces. Furthermore, neither the 10 ml nor 20 ml vial demonstrated a clear advantage over the other in terms of detecting VOCs at a higher abundance. These findings agree with work by others that an increase in headspace volume does not have an impact on analyte detection (Cho et al. 2003). However it may depend on sample matrix, as found in murine faeces and human faeces (Reade et al. 2014). A smaller headspace volume resulted in a higher yield of VOCs for murine, whereas for human faeces (and horse in the present work) an alteration to the headspace volume had little impact. The SPME theory suggests that decreasing the volume of headspace increases the chance of compounds being detected. A vial volume increase from 10 to 20 ml may not have provided a sufficient increase in headspace for a difference in VOCs to be seen in the present work. Greater numbers of VOCs may be extracted from samples with an increased exposure time or temperature (Reade et al. 2014). However an exposure time of 20 min and extraction temperature of 60 °C were optimal from those studied for both human and murine samples in our lab and therefore were not investigated here (Reade et al. 2014). An exposure time of longer than 20 min may also be impractical for large-scale studies requiring high-throughput analysis. Masses greater than 1000 mg were not investigated in 20 ml vials as significant differences between VOC numbers and abundances between 1000 and 2000 mg were not seen in 10 ml vials and it was thought that the SPME fibre may have reached its limit of absorbance at 1000 mg (Sect. 4.1). It can be concluded that both 10 and 20 ml vials are suitable for SPME-GC-MS analysis of 1000 mg horse faeces with little impact on the presence or abundance of VOCs obtained.

### Technical replicates

Technical replicates of 1000 mg of faeces clustered closely according to the horse sampled and all samples fell within a 95% confidence interval (Supplementary Fig. 1).Within each horse the CV of shared VOC peak areas were below 11%, indicating a good reproducibility of the method.

### Time and temperature of storage

Metabolic profiling of faeces using GC-MS analysis has been widely reported in numerous species. The processing of fresh samples is not always practical and therefore methods of preserving samples are necessary. However, the effect of storage length and temperature on faecal VOCs has received little attention (Deda et al. 2015). In the present work a higher number of compounds were found in samples stored at − 20 °C than fresh or − 80 °C. An increase in compounds after 1 month of freezing (− 80 °C) has been observed by others (Li et al. 2011). Chemical classification of VOCs revealed a large proportion of compounds in − 20 °C stored samples were esters. Furthermore VOCs accounting for the most variation in PC1 were largely esters (Supplementary Table 7). Fewer esters were seen in fresh samples and those stored at − 80 °C, indicating that the formation of esters is specific to − 20 °C storage. From this work it can only be speculated as to why esters were specific to storage at − 20 °C, possibly because at − 20 °C the sample may take longer to freeze and therefore esterification of acids continues compared to a sample frozen at − 80 °C. This phenomenon may be individual pony or storage vessel specific and requires further investigation. In human faeces it was observed that after 24 h of storage at − 20 °C there was no difference in VOC profile between that and a fresh sample (Gratton et al. 2016). Species differences are evident in preparation steps for metabolomics analysis (Reade et al. 2014). It is likely these differences are because of the differing VOC profiles between species, largely attributed to diet and intestinal microbiota. Unique storage conditions may be required for different types of sample. It was interesting to note that the percentage of VOCs shared between the fresh sample and samples stored at − 80 °C for 6 and 12 months decreased. An example includes benzene, (1-methylethyl)- which was present in a fresh sample and a sample stored at − 80 °C for 1 week, but was not present in any − 80 °C samples after 6 or 12 months. Whereas 1-octen-3-ol was not present in the fresh sample but was detected in − 80 °C samples after storage of 1 week, 6 months and 12 months. From this work it is difficult to determine why VOCs were lost or gained after storage as we still know very little of the impact of freezing on VOCs (Berkhout et al. 2016). We speculate the loss or gain of VOCs may be attributed to the material a sample is stored in (Mochalski et al. 2009) or the effect of freeze-thawing on microorganisms present in faeces (Achá et al. 2005). A limitation of this investigation is that a faecal sample was obtained from one animal. However, to the authors’ knowledge this is the first work attempting to address the effect of long-term storage at varying temperatures on equine faecal VOCs and has highlighted the importance of these factors in experimental design. Based on this work a sample should be stored at − 80 °C and analysed within 1 week to most resemble a fresh sample. Further work is necessary to confirm these findings in larger sample sizes and in other species.

## Overall discussion

An optimal method for the preparation of samples for metabolomics is essential for achieving accurate and reproducible results. The preparation method for metabolic profiling differs between techniques selected, sample matrix and species. This work is the first of its kind working towards a robust method to extract VOCs from equine faeces using HS-SPME-GCMS. It was found that aliquots of 1000 mg in 10 or 20 ml SPME headspace vials were optimal. Volatile organic compounds should ideally be extracted using a DVB-CAR-PDMS SPME fibre. Faecal samples for VOC analysis to be stored at − 80 °C up to 12 months, where this is not possible all samples should be stored for the same length of time at the same temperature.

One of the main limitations of this work are the low number of replicates, particularly for the investigation of the storage of samples. A larger sample size may be better equipped to account for the influence of individual differences including breeds of horse, diet, age, effect of anthelmintic treatment etc. which may influence the faecal metabolome of the horse and hence the optimal method required for extraction of VOCs. However very few of these factors and their impact on the equine faecal VOC metabolome have been studied. The large gaps in measurement variables chosen here act only as a starting point and more precise intervals should feature in future work. Interactions between experimental factors were not investigated here. The reasons for this are that it was likely that saturation of the SPME fibre or over-loading of the instrument had started to occur at 1000 mg and there was no difference when this mass of sample was placed in a larger vial. Furthermore the CAR-PDMS fibre did not produce any advantage over 1000 mg in a 10 ml vial therefore we did not investigate any further sample masses with this fibre coating. A number of quality control techniques were applied in this work including the regular testing of laboratory air during analysis and blanks were run between samples to prevent carry-over. Samples were analysed in triplicate and the order of running samples was computer randomised. However, techniques to account for systematic variation e.g. the use of internal standards or pooled samples, were not explored here. As reviewed by (Dudzik et al. 2018) it is very important that robust quality control techniques are made available and should be explored in future method development work for extracting VOCs from equine faeces.

## Conclusions

This work is a starting point for working towards a standardised method will allow comparisons to be made between future equine faecal GCMS based studies. Future work should involve larger sample sizes, consider individual characteristics and explore more precise measurement variables and quality control techniques.

## Electronic Supplementary Material

Below is the link to the electronic supplementary material.

Supplementary material 1 (XLSX 78 KB)

Supplementary material 2 (XLSX 93 KB)

## References

[CR1] Achá SJ, Kühn I, Mbazima G, Colque-Navarro P, Möllby R (2005). Changes of viability and composition of the Escherichia coli flora in faecal samples during long time storage. Journal of Microbiological Methods.

[CR2] Aggio R, Villas-Bôas SG, Ruggiero K (2011). Metab: An R package for high-throughput analysis of metabolomics data generated by GC-MS. Bioinformatics.

[CR3] Amann A, Costello BDL, Miekisch W, Schubert J, Buszewski B, Pleil J (2014). The human volatilome: Volatile organic compounds (VOCs) in exhaled breath, skin emanations,urine, feces and saliva. Journal of Breath Research.

[CR4] Arthur CL, Pawliszyn J (1990). Solid phase microextraction with thermal desorption using fused silica optical fibers. Analytical Chemistry.

[CR5] Bergman EN (1990). Energy contributions of volatile fatty acids from the gastrointestinal tract in various species. Physiological Reviews.

[CR6] Berkhout D, Benninga M, van Stein R, Brinkman P, Niemarkt H, de Boer N (2016). Effects of sampling conditions and environmental factors on fecal volatile organic compound analysis by an electronic nose device. Sensors.

[CR31] Bianchi, F., Dall’Asta, M., Del Rio, D., Mangia, A., Musci, M., & Scazzina, F. (2011). Development of a headspace solid-phase microextraction gas chromatography–mass spectrometric method for the determination of short-chain fatty acids from intestinal fermentation. *Food Chemistry*, *129*, 200–205.

[CR7] Cavalli JF, Fernandez X, Lizzani-Cuvelier L, Loiseau AM (2003). Comparison of static headspace, headspace solid phase microextraction, headspace sorptive extraction, and direct thermal desorption techniques on chemical composition of French olive oils. Journal of Agricultural and Food Chemistry.

[CR8] Cho DH, Kong SH, Oh SG (2003). Analysis of trihalomethanes in drinking water using headspace-SPME technique with gas chromatography. Water Research.

[CR32] Couch Robin D., Navarro Karl, Sikaroodi Masoumeh, Gillevet Pat, Forsyth Christopher B., Mutlu Ece, Engen Phillip A., Keshavarzian Ali (2013). The Approach to Sample Acquisition and Its Impact on the Derived Human Fecal Microbiome and VOC Metabolome. PLoS ONE.

[CR9] Daly K, Proudman CJ, Duncan SH, Flint HJ, Dyer J, Shirazi-Beechey SP (2012). Alterations in microbiota and fermentation products in equine large intestine in response to dietary variation and intestinal disease. The British Journal of Nutrition.

[CR10] Deda O, Gika HG, Wilson I, Theodoridis GA (2015). An overview of fecal sample preparation for global metabolic profiling. Journal of Pharmaceutical and Biomedical Analysis.

[CR33] Dixon Emma, Clubb Cynthia, Pittman Sara, Ammann Larry, Rasheed Zeehasham, Kazmi Nazia, Keshavarzian Ali, Gillevet Pat, Rangwala Huzefa, Couch Robin D. (2011). Solid-Phase Microextraction and the Human Fecal VOC Metabolome. PLoS ONE.

[CR11] Dudzik D, Barbas-Bernardos C, García A, Barbas C (2018). Quality assurance procedures for mass spectrometry untargeted metabolomics: A review. Journal of Pharmaceutical and Biomedical Analysis.

[CR12] Garner CE, Smith S, de Lacy Costello B, White P, Spencer R, Probert CSJ (2007). Volatile organic compounds from feces and their potential for diagnosis of gastrointestinal disease. FASEB Journal.

[CR13] Garner CE, Smith S, Elviss NC, Humphrey TJ, White P, Ratcliffe NM (2008). Identification of campylobacter infection in chickens from volatile faecal emissions. Biomarkers.

[CR14] Gianelli MP, Flores M, Toldra F (2002). Optimisation of solid phase microextraction (SPME) for the analysis of volatile compounds in dry-cured ham. Journal of the Science of Food and Agriculture.

[CR15] Gratton J, Phetcharaburanin J, Mullish BH, Williams HRT, Thursz M, Nicholson JK (2016). Optimized sample handling strategy for metabolic profiling of human feces. Analytical Chemistry.

[CR16] Ishizaka S, Matsuda A, Amagai Y, Oida K, Jang H, Ueda Y (2014). Oral administration of fermented probiotics improves the condition of feces in adult horses. Journal of Equine Science.

[CR17] Li JV, Saric J, Wang Y, Keiser J, Utzinger J, Holmes E (2011). Chemometric analysis of biofluids from mice experimentally infected with Schistosoma mansoni. Parasites & Vectors.

[CR18] Mani V, Pawliszyn J (1999). Properties of commercial SPME fibre coatings. Applications of solid phase microextraction.

[CR19] Marcobal A, Kashyap PC, Nelson TA, Aronov PA, Donia MS, Spormann A (2013). A metabolomic view of how the human gut microbiota impacts the host metabolome using humanized and gnotobiotic mice. ISME Journal.

[CR20] Milinovich GJ, Trott DJ, Burrell PC, Croser EL, Al Jassim RA, Morton JM (2007). Fluorescence in situ hybridization analysis of hindgut bacteria associated with the development of equine laminitis. Environmental Microbiology.

[CR21] Mochalski P, Wzorek B, Śliwka I, Amann A (2009). Suitability of different polymer bags for storage of volatile sulphur compounds relevant to breath analysis. Journal of Chromatography B.

[CR22] Povolo M, Contarini G (2003). C omparison of solid-phase microextraction and purge-and-trap methods for the analysis of the volatile fraction of butter. Journal of Chromatography A.

[CR23] Proudman CJ, Hunter JO, Darby aC, Escalona EE, Batty C, Turner C (2014). Characterisation of the faecal metabolome and microbiome of Thoroughbred racehorses. Equine Veterinary Journal.

[CR24] Reade S, Mayor A, Aggio R, Khalid T, Pritchard D, Ewer A (2014). Optimisation of sample preparation for direct SPME-GC-MS analysis of murine and human faecal volatile organic compounds for metabolomic studies. Journal of Analytical & Bioanalytical Techniques.

[CR25] Saric J, Wang Y, Li J, Coen M, Utzinger J, Marchesi JR (2008). Species variation in the fecal metabolome gives insight into differential gastrointestinal function. Journal of Proteome Research.

[CR26] Stavert JR, Drayton BA, Beggs JR, Gaskett AC (2014). The volatile organic compounds of introduced and native dung and carrion and their role in dung beetle foraging behaviour. Ecological Entomology.

[CR27] Ng JSY, Ryan U, Trengove R, Maker G (2012). Development of an untargeted metabolomics method for the anlysis of human faecal samples using *Cryptosporidium*-infected samples. Molecular & Biochemical Parasitology.

[CR28] Sumner LW, Amberg A, Barrett D, Beale MH, Beger R, Daykin CA (2007). Proposed minimum reporting standards for chemical analysis. Metabolomics.

[CR29] Turner C, Batty C, Escalona E, Hunter J, Proudman C (2013). The use of SIFT-MS in profiling the faecal volatile metabolome in horses with colic:A pilot study. Current Analytical Chemistry.

[CR30] Xia J, Mandal R, Sinelnikov IV, Broadhurst D, Wishart DS (2012). MetaboAnalyst 2.0-a comprehensive server for metabolomic data analysis. Nucleic Acids Research.

